# A protocol for training group-housed rhesus macaques (*Macaca mulatta*) to cooperate with husbandry and research procedures using positive reinforcement

**DOI:** 10.1016/j.applanim.2017.08.006

**Published:** 2017-12

**Authors:** Caralyn Kemp, Harriet Thatcher, David Farningham, Claire Witham, Ann MacLarnon, Amanda Holmes, Stuart Semple, Emily J. Bethell

**Affiliations:** a Centre for Research in Brain and Behaviour, School of Natural Sciences and Psychology, Liverpool John Moores University, L3 3AF, UK; b Medical Research Council Harwell Unit, Centre for Macaques, Salisbury, Wiltshire, SP4 0JQ, UK; c Institute of Neuroscience, Newcastle University, Newcastle-upon-Tyne, NE1 7RU, UK; d Centre for Research in Evolutionary, Social and Interdisciplinary Anthropology, University of Roehampton, London, SW15 4JD, UK

**Keywords:** 3Rs, Group training, Macaque, Positive reinforcement training, Primates, Stationing

## Abstract

There has been increased recognition of the 3Rs in laboratory animal management over the last decade, including improvements in animal handling and housing. For example, positive reinforcement is now more widely used to encourage primates to cooperate with husbandry procedures, and improved enclosure design allows housing in social groups with opportunity to escape and avoid other primates and humans. Both practices have become gold standards in captive primate care resulting in improved health and behavioural outcomes. However, training individuals and social housing may be perceived as incompatible, and so it is important to share protocols, their outcomes and suggestions for planning and improvements for future uptake. Here we present a protocol with link to video for training rhesus macaques (*Macaca mulatta)* housed in single-male – multi-female breeding groups to sit at individual stations in the social enclosure. Our aim was that the monkeys could take part in welfare-related cognitive assessments without the need for removal from the group or interference by group members. To do this we required most individuals in a group to sit by individual stations at the same time. Most of the training was conducted by a single trainer with occasional assistance from a second trainer depending on availability. We successfully trained 61/65 monkeys housed in groups of up to nine adults (plus infants and juveniles) to sit by their individual stationing tools for >30 s. Males successfully trained on average within 30 min (2 training sessions); females trained on average in 1 h 52 min ± 13min (7.44 sessions), with rank (high, mid, low) affecting the number of sessions required. On average, dominant females trained in 1 h 26 min ± 16 min (5.7 sessions), mid ranked females in 1 h 52 min ± 20min (7.45 sessions), and subordinate females took 2 h 44 min ± 36 min (10.9 sessions). Age, group size, reproductive status, temperament, and early maternal separation did not influence the number of sessions a monkey required to reach criterion. We hope this protocol will be useful for facilities worldwide looking to house their animals in naturalistic social groups without impacting on animal husbandry and management.

## Highlights

• We present a protocol for station-training adult rhesus macaques in social groups of 2–9 adults.
• 61/65 monkeys successfully trained to sit by individual targets.
• All males trained in 2 × 15 min training sessions.
• High rank females trained in 6 × 15 min sessions; low rank females: 11 × 15 min.
• Dominance rank was the only predictor of time taken to train.

## 1 Introduction

With the increased recognition of the 3Rs in research (NC3Rs, 2006, Prescott, 2010, Russell and Burch, 1959), training laboratory primates to cooperate with animal management and research procedures has become a key welfare refinement (Bloomsmith et al., 1998, Coleman et al., 2008, LASA/MRC, 2004, Laule et al., 1996, Laule et al., 2003, NC3Rs, 2015, Perlman et al., 2010, Perlman et al., 2012, Prescott and Buchanan-Smith, 2007, Reinhardt, 1997, Schapiro et al., 2003, Schapiro et al., 2005). However, emphasis on housing conditions that fulfil animals’ physical and social needs can result in perceived conflicts between colony management practices and animal welfare (Prescott and Buchanan-Smith, 2007). It is therefore important to document and share training protocols and outcomes from facilities embracing the 3Rs in their management plans, so that means of best practice can be shared and developed further.

Training animals teaches them that their behaviour has consequences, and positive reinforcement training (PRT) is particularly recommended from a welfare perspective because it encourages voluntary participation for positive outcomes (Bassett and Buchanan-Smith, 2007, Prescott and Buchanan-Smith, 2007, Westlund, 2015). The theory underlying PRT has been well described elsewhere (e.g. Bloomsmith et al., 2007, Laule and Whittaker, 2001, Laule and Whittaker, 2007, Schapiro et al., 2005, Westlund, 2015) and we give key terms and definitions in Table 1. There is widespread agreement that opportunity for choice and control afforded by PRT not only has direct welfare benefits (Bassett and Buchanan-Smith, 2007, Buchanan-Smith and Badihi, 2012) but may also improve the quality of research data arising from use of animal models (e.g. Lambeth et al., 2006, Prescott et al., 2010). Furthermore, PRT can provide a valuable colony management tool with time and money savings, resulting from a cooperative relationship built on trust between trainer and trainee (Jennings et al., 2009).

**Table 1:** Glossary.

Term	Definition
Positive reinforcement	The occurrence of a behaviour is increased as it results in a reward (e.g. food)
Negative reinforcement	The occurrence of a behaviour is increased as it results in removal of an aversive stimulus (e.g. capture net)
Positive punishment	The occurrence of a behaviour is decreased as it results in the appearance of an aversive stimulus (e.g. verbal ‘no’)
Negative punishment	The occurrence of a behaviour is decreased as it results in removal of a reward (e.g. it results in a ‘time out’)
Shaping	also ‘successive approximation’. A desired behaviour (such as ‘hold target for 30 s’) is broken down into successive stages (approach target, touch target, hold target, stay by target).
Bridge	A type of ‘conditioned reinforcer “or ‘secondary reinforcer”. An initially unfamiliar stimulus (such as the “click” of a hand-held clicker or a verbal cue such as ‘good’) is repeatedly paired with a primary reinforcer so that it becomes a positive reinforcer through association. Specifically, a bridging stimulus can be produced exactly at the moment the animal performs a desired behaviour, therefore creating a bridge between performing the behaviour and receiving the primary reinforcer (e.g. food).

While PRT requires an initial time investment, evidence suggests this is small compared to the long term time savings afforded by animals who calmly and efficiently participate in husbandry and research procedures due to reduced stress, and faster and improved performance (Lambeth et al., 2006, Perlman et al., 2012, Reinhardt et al., 1990, Westlund, 2015). Well trained animals are more likely to participate in further, more advanced, training procedures, and may be more likely to successfully participate in more cognitively demanding research protocols (Jennings et al., 2009, Westlund, 2015). Reduced stress levels contribute to improved health and reproductive outcomes (e.g. Shively et al., 2005, Capitanio et al., 1998). We also suggest that implementing standardised group-training protocols across facilities, and especially at breeding centres and in younger animals, may provide a useful mechanism for minimising relocation stress in animals transferred between facilities (e.g. Honess et al., 2004). As animals are often transferred from breeding facilities to research centres, training familiarity may help them adjust more readily to new environments with unfamiliar staff.

There are a number of published surveys of facility-wide practices and staff perceptions (e.g. Prescott and Buchanan-Smith, 2007, Perlman et al., 2012) and some published protocols for training (e.g. Westlund, 2015, Laule et al., 2003). However, there are very few studies detailing group-level training protocols together with data on training success rates. Of the published studies, descriptions of training outcomes for primates typically involve relatively small numbers of individually trained animals (e.g. Bloomsmith et al., 1994, Reinhardt, 1997, Ward and Melfi, 2013), and animals in single or pair housing (Clay et al., 2009, Coleman et al., 2008, Fernstrom et al., 2009, Laule et al., 1996, Reinhardt, 1997, Reinhardt et al., 1990). The training of primates in groups (n > 3) tends to cover three categories of behaviour: collective behaviour, individual behaviour, and cooperative behaviour. PRT of collective behaviour involves training a group to work together to achieve a goal, with all group members performing the same behaviour, such as moving from one part of their enclosure to another (e.g. Bloomsmith et al., 1998; Veeder et al., 2009). Individuals within a group can also be trained, one at a time, to perform a task (e.g. Fagot et al., 2014; Stone et al., 1994) by simply encouraging the target animal to one location of the enclosure and ignoring any other group members who might approach to investigate. The training of cooperative behaviour is usually focused on group management, such as cooperative feeding (Bloomsmith et al., 1994, Whittaker, 2005, in which dominant animals are reinforced for allowing lower-ranked conspecifics access to desirable resources. Training animals in groups therefore requires staff to be sensitive to group dynamics and it can be daunting for staff to initiate training efforts when the primates are not typical research subjects (i.e. training naïve) and live in large groups, such as in a breeding facility or zoological institution (Westlund, 2015). The initiation and objective success of group training programs with larger numbers of animals therefore requires greater documentation and validation (Perlman et al., 2012; Prescott and Bucahanan-Smith, 2007), especially for animals in high-welfare housing conditions where the opportunity to move freely may be perceived as a barrier to staff initiating and maintain training.

Here we present the training protocol and training outcomes for group-housed rhesus macaques (*Macaca mulatta*) taking part in an NC3Rs-funded research project (NC/L000539/1) investigating cognitive measures of psychological wellbeing. Our research was conducted within a breeding facility where macaque group sizes ranged from two to 11 adults, plus infants and juveniles. The methodology for the research project required the adult female macaques to remain by a stationing tool so that they could be individually presented with stimuli, and their responses filmed by a fixed camera (Bethell et al., 2015, Szott, 2015, Thatcher, 2016). For both scientific and welfare purposes, it was important that the macaques remained within their social group during testing and that we minimised any actions that might cause stress. To this end, we planned to train all adults within each group to allow control over the group as a whole. The trainers (CK as primary trainer with later assistance from HT) had to divide their duties during the research stage and so it was essential that the monkeys could be managed as a group by one trainer. This paper details the training methods used and the outcomes, including best predictors of training success. We hope this will provide a useful protocol for other facilities to encourage training of animals to engage in routine procedures without the need for removal from the social group.

## 2 Methods

### 2.1 Ethics

The research program and training plans were formulated in discussion with the facility Home Office Inspector (Nov 2011) and subsequently approved by Roehampton University Ethics Committee (approval #LSC 14/113).

### 2.2 Animals and housing

Sixty-five adult rhesus macaques (65 female, 9 male; age range 29–220 months) housed as part of the breeding stock at the Medical Research Council’s Centre for Macaques (MRC-CFM) took part in the training. The MRC-CFM is licenced by the Home Office to breed macaques for provision to UK facilities. MRC-CFM works in strict accordance with the NC3Rs guidelines (NC3Rs, 2006). Images of the facility's primate accommodation are available to view in the NC3Rs guidelines (NC3Rs, 2006) and on the NC3Rs macaque website (NC3Rs, 2015) as examples of good practice in animal housing and enrichment.

Monkeys were housed in 11 social groups, eight of which consisted of one adult male and breeding females, with infants and juveniles, and three of which contained only adult females. Groups were selected for training if they contained females who would later take part in a research study of cognitive markers of wellbeing (Bethell et al., in prep.). A number of life history variables were recorded for each monkey including sex, age and group size. For females we additionally noted: reproductive status (pregnant, dependent offspring, neither or both: these were obtained from visual inspection and retrospectively by working back from timings of births); rank within the social hierarchy (high, mid or low); temperament (ranging from affiliative to aggressive, described in more detail below); and whether they had been removed from the mother earlier than 1 year of age (early maternal separation as a proxy for early life stress).

Rhesus macaques have a linear hierarchy based on female relatedness and relationships (deWaal and Luttrell, 1985, Jackson and Winnegrad, 1988). We determined the rank of each female within her group through consultation with facility staff and through observation of displacements, direction of aggression, and vigilance during the initial habituation phase. Two researchers (CK and HT) conducted separate assessments and then compared for accordance, the result of which shows that the hierarchal position of each female was clearly defined. Confidence and wariness were clear signals of status, with dominant females typically approaching the trainer early in the process. Who was wary of whom, as well as aggressive events between females, also helped determine rank. High ranking females tended to dominate priority locations, especially near the breeding male, and would sit on the middle level of the caging. Very low ranked females utilised the bottom level, stayed near hatchways, and were quick to flee when more dominant animals approached them. Once the linear order of the females for each group was determined, we calculated each animal’s relative rank within their group. Typically, we assigned the top 2–3 females as high ranking, the bottom 1–2 females as low ranking, and all others as mid-ranked, and adjusted this according to the relative numbers in the group and exertion of dominance by the top female.

Temperament was classified by CK based on three categories of observations (Table 2): focal animal behavioural observation in the social group; behavioural responses towards and eagerness to approach trainers during habituation and training sessions (ie., confident to approach and cooperate or wary and uncooperative); and behavioural interactions with group members during habituation and training (i.e. willingness to let others receive rewards, how closely subordinates were allowed to sit, aggressive and submissive behaviours). From these observations, we were interested in consistent characteristics that indicated whether an animal was predominantly (more than 60% of the time) ‘affiliative and cooperative’, ‘aggressive and uncooperative’ or ‘predominantly neither’ (that is, fitting into neither category clearly).

**Table 2:** Behavioural categories used to describe temperament as either affiliative/cooperative, aggressive/uncooperative, or mixed, when observations were not predominantly (>60%) one or the other.

Context for observation	Interaction with…	Temperament category	Description of observations
During habituation and station training sessions	Trainer	Affiliative/cooperative	Approaches training staff quickly when indicated.
			Allows other adult females to be trained.
			Does not snatch treats and run away.
			Remains in cage room consistently.
			Utilises dominant locations. Unfazed by presentation of stationing tools – quick to investigate (within 2 min of first presentation).
		Aggressive/uncooperative	Threatening trainer during sessions.
			Snatches treats and runs away.
			Spends a lot of time in hatchway or play room.
			Utilises lower levels of caging area.
			Nervous about stationing tools – not quick to investigate (more than 2 min or multiple sessions).
	Conspecifics	Affiliative/cooperative	Allows other adult females to receive treats without challenging.
			Allows at least one other adult female to sit within 1 m on the same horizontal level.
		Aggressive/uncooperative	Threatens adult females when they are offered treats.
Focal observation of animals in the social group	Conspecifics	Affiliative/cooperative	Grooming other adult female in group.
			Sitting closely (bodily contact) with other adult female/s.
		Aggressive/uncooperative	Displacing an adult female.
			Attacking, biting, hitting, chasing other adult female/s.

Each group had access to a free-roaming room (3.35 m × 8.04 m × 2.8m) and an adjacent cage area (1.5 m × 6.12 m × 2.8m), accessible through hatches, with a minimum total space of 3.5 m^3^/breeding animal in the largest groups. Each free-roaming room had a large bay window at one end facing outdoors and allowing a natural day-night cycle. At the other end of each room was an internal window into the hallway used by staff. Internal windows were fitted with movable mirrors so that monkeys could manipulate the mirrors to view activities along the corridor. Rooms were furnished with wooden platforms and poles (horizontal, vertical, diagonal), fire hose, ladders, plastic horse jumps and saddle racks, PVC piping, plastic barrels and balls, and small plastic blocks attached to structures or walls. The floor was covered with a deep layer of straw and shavings. All rooms were temperature controlled (20 °C ± 5) with humidity at 55% ± 10.

Animals were free to move between the room and cage area at all times during training and at no point were the hatches used to retain animals. Adjacent groups were able to see and hear each other from the cage area, but there was no possibility for physical contact. All training took place in the cage area, with open access to the free-roaming room at all times.

The macaques were fed twice daily by scatter feed, morning and afternoon, with sufficient food to last for a 24 h period. The diet varied daily and included a dried forage mix (cereal, peas, beans, lentils etc.), a range of fruit and vegetables, bread and boiled eggs. Water was available ad libitum in both the room and cage area.

### 2.3 Training protocol

**Fig. 1:**
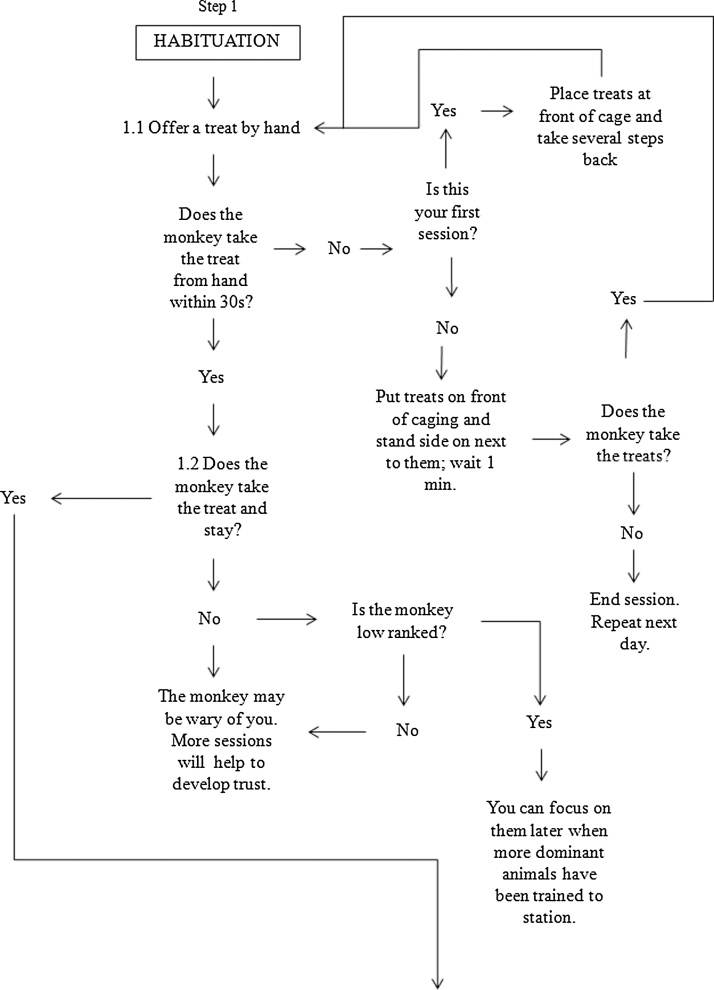

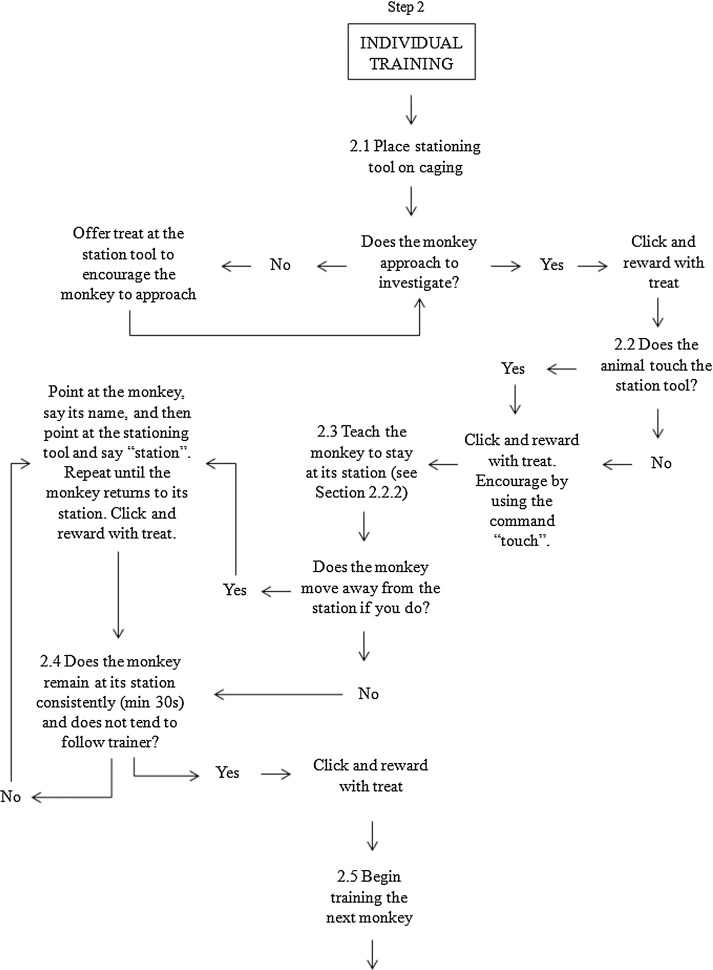

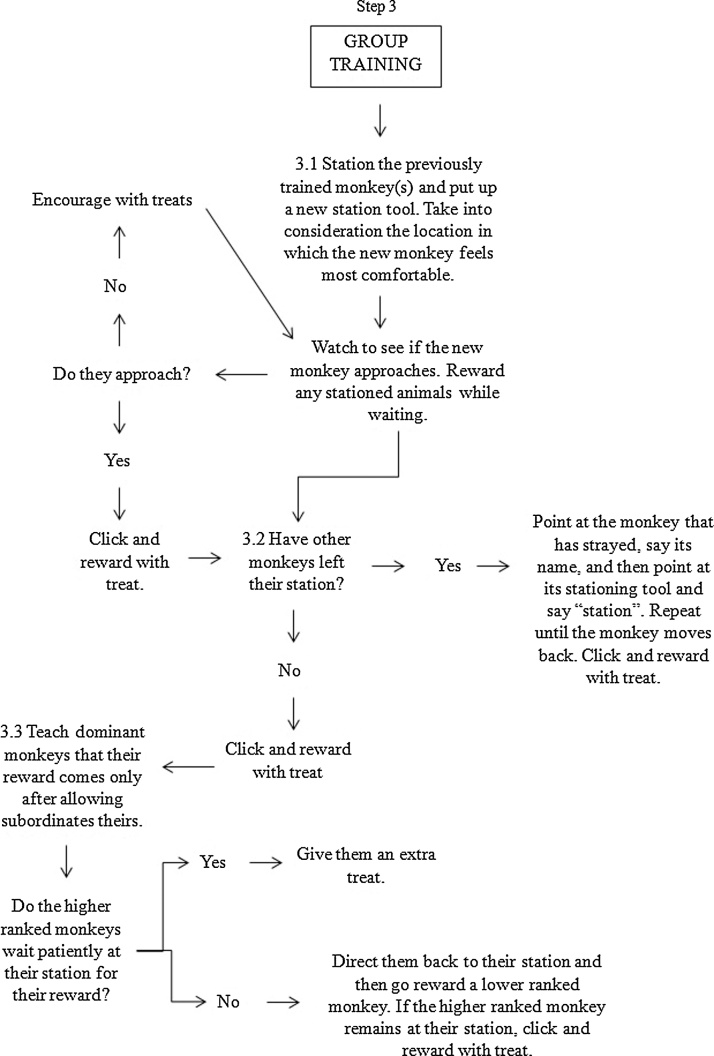
Flowchart for training steps. This flowchart details the methodology and stepwise decision making process used to train the macaques, beginning with habituation (Step 1), moving to training an individual monkey (Step 2), to training the animals as a group (Step 3).

The training protocol is shown in Fig. 1. Video of group target training may be viewed at: https://www.mrc.ac.uk/research/facilities-and-resources-for-researchers/mrc-centre-for-macaques/habituation-and-training/. The key aims of training were to a) establish clear and consistent signals for rewards and b) develop a relationship of trust that the trainer will behave consistently.

#### 2.3.1 Habituation

Prior to training, all groups went through a period of habituation to familiarise them with the trainers and the clicker device which used as a secondary reinforcer to ‘bridge’ between the moment of the desired behaviour and reward (see Table 1). CK and HT were not members of care staff at the facility, and monkeys therefore first needed to be habituated to their presence (Fig. 1, Step 1). At the start of the study, three habituation-only sessions (5–10 min in length) were conducted for each group once on each of three separate days within a 1 week window. During a habituation session, small pieces of preferred food treats (peanuts and raisins) were offered in the caged area (Fig. 1, Step 1.1). Treats were small to prevent satiation and over-feeding. All monkeys were offered and encouraged to take treats from the trainer’s hand. When there was reluctance to do so the treats were placed on the cage bars to entice the monkey to move forward. If a monkey was particularly nervous, the trainer would initially step back when the monkey approached the front of the cage to encourage confidence to move forward for treats. A clicker device was sounded at the moment when the monkey took a treat, accompanied with the verbal reinforcement of “good boy/girl *name*”. Verbal commands were given to assist the monkeys in developing a positive association for trainers and researchers saying their name, and the use of verbal and clicker cues together was considered to enhance opportunity for learning (e.g. Westlund, 2015, Fernstrom et al., 2009).

Once one or two monkeys were comfortable coming forward for treats and staying at the front of the cage to feed (Fig. 1, Step 1.2), training sessions began (Fig. 1, Step 2). Training the food-dominating monkeys to station first allowed us to manage the group most effectively. By stationing these animals first, they learned to cooperate and this allowed us to then focus on other group members, encourage them to come forward and train them individually in the group setting.

#### 2.3.2 Training the first individual

All training sessions were kept to a maximum of 15 min. One training session was conducted per day, as this had previously been found to be the most efficient frequency for the successful training of macaques (Fernstrom et al., 2009). Training was conducted with a focus on using positive reinforcement for desired behaviours: in this case holding onto a target for stationing. The clicker was used as a secondary reinforcer, or “bridge”, with treats (peanut or raisin pieces) as the primary reinforcer. As the monkeys became more comfortable with the presence of the researchers and taking treats by hand, the clicker was used as a bridge, and activated prior to or instead of the treat. Generally, peanuts and raisins were given out on different days but some monkeys had a preference and would not cooperate for the other treat, and so efforts were made to adapt to individual preferences

Training proceeded in the same manner for each individual in the group (Fig. 1, Step 3). In the breeding groups, training was always first conducted with the breeding male. Although they were not tested as part of the overall research program, it was important to train them to station and keep out of the way of the females who were taking part in the research. This discipline reduced the likelihood of the male interrupting training and testing sessions with the females, in particular the lower ranked females, in order to steal their treats. The males were also trained to sit when at their station (the females tended to sit at their station automatically) using the verbal command “sit” and a corresponding hand gesture. We observed that when trained to sit, males were less likely to move away from their station.

**Fig 2:**
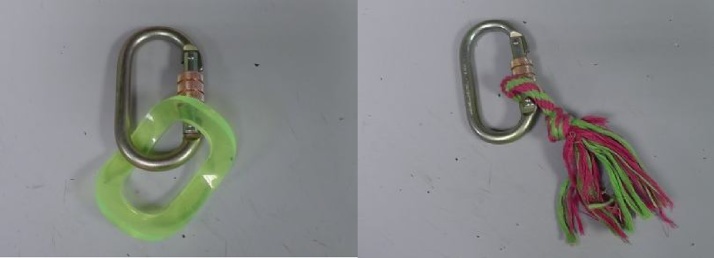
Examples of stationing tools. Each macaque was assigned a unique stationing tool that they learned to hold.

Each monkey was assigned an individual stationing tool (Fig. 2). Station tools were designed to be strong, durable, safe and distinctive in appearance; we used durable dog toys attached to carabiners and then clipped to the caging. The monkeys were given the opportunity to investigate the station tool. When the male approached his assigned stationing tool, he was rewarded with a click, a treat, and a verbal cue of “good boy *name*”. This behaviour was gradually shaped over time so that he was only ever rewarded if he sat next to and was touching his station tool for progressively longer periods of time. If the monkey had shown interest in the station tool and approached and received treats, but moved away during the training, the trainer would walk over to the target monkey, point at them and say their name, and then walk to the station tool, point at it and use the verbal cue of “station”. This would be repeated as often as necessary within the limitations of the training session so that the monkey would associate a particular station with themselves. If the monkey touched the stationing tool (Fig. 1, Step 2.2), they were also rewarded. If the monkey did not touch the stationing tool, we would put food on the carabiner or push the carabiner in between the bars towards the monkey to encourage exploration and we found that many macaques responded to this action by reaching out to the carabiner if only to push it back out – this touching was always rewarded.

The aim of our training protocol was for the monkey to hold on to some aspect of the stationing tool to encourage them to remain in one location and not follow the trainer (Fig. 1, Step 2.4). It was therefore necessary that touching became holding. To this end, the length of time the monkey had to be in contact with the station before being rewarded steadily increased from a brief touch to up to 30 s (i.e. shaping, Laule et al., 2003). The verbal cue “hold” was used. With the longer periods of holding, we used the clicker to reinforce the behaviour but did not give a food reward until the target time period had been achieved. Once an animal had reached the threshold of 30 s of continuous holding, we found that most macaques would continue holding throughout training and testing.

Some macaques would not touch their station at all, despite repeated efforts, but would remain at it. This was fine for our testing needs, so long as the monkey consistently remained at its station (Fig. 1, Step 2.4), and so we did not continue pushing these animals to touch the tool itself. However, we found that some of these animals would much later (typically months after learning to sit by their station) start exploring the stationing tool and touch it. This was then rewarded and encouraged as described in Fig. 1 (Steps 2.2–2.4).

At the end of the training session, the verbal cue of “all done”, with a corresponding waving hand gesture, was used before the station tool was removed. This cue was used to signify to the macaques that the training session was over and that no more signals or rewards were coming. Although unique cues signalling the start and end of training sessions have not been assessed within the literature, there is debate amongst trainers regarding their usefulness (see Pryor, 1999). One thought is that they are important for the animals to understand when they are in a ‘training’ context as opposed to other contexts (e.g. cleaning or feeding). This may speed up the learning process, as it helps animals to distinguish disruptions to training due to extraneous factors from the intended completion of a session. We also did not test whether or not the signal was necessary. However, we felt it was useful, given the large number of macaques per trainer, for an end signal to be used so that the animals would learn that even when the trainer was not working directly with them, the session was continuing and therefore they should remain at their station in order to receive a reward.

#### 2.3.3 Training the group

Once the first animal, usually the breeding male when one was present, had learned to station for at least 30 s, we began training the next individual (Fig. 1, Step 2.5), usually the dominant female. We started a training session by stationing the first animal who had been trained. Once the first animal had been stationed, the trainer moved away and attached a new stationing tool to the caging at a distance at least out of arms’ reach (Fig. 1, Step 3.1). Through trial and error we learnt which animals could be stationed near each other without aggression and which needed to be kept well apart; we also utilised different heights in the cage area, and adapted to individuals’ preferences for positioning, especially for the larger groups. Low ranked animals, in particular, tended to prefer to be in a position where they could view the breeding male (or more dominant females) but were not on the same level and therefore had a quick escape route if necessary. It helped, in some groups, to insert dividing panels into the caging to act as visual barriers between particular group members; however, this method was used sparingly as use of dividing panels can signal multiple outcomes (including negative events such as veterinary inspections), and it was necessary to spend time habituating the animals to the panels being put in.

Initially, the first monkey to be trained would typically follow the trainer as they started training the second animal (Fig. 1, Step 3.2) and so it was necessary to walk them back to their own station, using the finger point hand gesture, starting from the animal (with the verbal cue of their name) and moving to the station (with the verbal cue of “station”). Over time we would stop rewarding with food when they returned to their station. At this point in training, only remaining at the station without interruption for longer durations was rewarded. Ignoring an animal who had learnt this rule but still left their station to follow the trainer would result in the monkey returning to their own station without command. This would be rewarded with a click and verbal cue of “good girl/boy *name*” but no food.

It was necessary for the trainer to be aware of the group dynamics as the training progressed, rather than remaining solely focused on any one particular individual. The trainer could only focus on each new animal for a short period of time before it was necessary to reward the previously trained animals. However, the time between rewards for the trained animals increased over time so that attention could be paid to each new monkey being trained. This also meant that higher ranked macaques learnt that they were only rewarded if they allowed lower ranked animals to receive their treats first; this was essential to reduce aggression. Once trained, the dominant animals were given larger rewards than the subordinates. In larger groups, it was helpful (although not essential) to have a second trainer present so that one person could focus on maintaining the already trained animals in position while the other trainer focused on a new trainee, or on training two new macaques simultaneously while the first person reinforced the rest of the group together.

The process of training individuals within a social group was typically oriented around the hierarchy, with the lowest ranked animals coming forward for training last in a group. It was important that, as the number of trained animals increased, the trainer did not leave the animal being trained to reinforce all the other monkeys who were waiting; this would be too long a disruption to the training. Instead, the trainer would reward only two or three animals before returning to the trainee and then reward a different two or three monkeys at the next opportunity. Importantly, the breeding male was rewarded more often than the females, especially when he was known to be particularly food-oriented or aggressive.

At the start of a training session with multiple trained animals, the trainer would always put the stationing tools up in the same order, starting with the breeding male, the dominant female and then working through animals down the hierarchy (typically in the order of training). At the end of the session, the station tools were removed in the reverse order. Each animal was given the “all done” cue individually. The criterion for successful training was defined as stationing for >30 s while we worked with other animals in the group. Once an animal reached criterion for successful training we viewed subsequent sessions as ‘maintenance’ sessions. We had 60 days to train monkeys prior to the onset of the cognitive study for which they were being trained to station.

#### 2.3.4 Dealing with undesired behaviours

Although our training focused on positive reinforcement methods, the trainer also gave some indication when an undesired behaviour had occurred. PRT standards recommend ignoring the behaviour by not providing a reward and encouraging extinction of the behaviour (Pryor, 1999). However, in our protocol, we occasionally used the word “no” to indicate an unwanted behavioural response from the monkeys and no click/treat was given. This was especially useful when two trainers were present to coordinate between us. If a monkey persistently gave an undesired behaviour (such as moving away from the stationing tool) and the use of the previously learned verbal or gestural cues for the desired activity was ignored, the trainer would hold out their hands with palms open (to signal no food), and then turn their back (i.e. a “time out”; Prescott et al., 2005).

### 2.4 Statistical analysis

Data on training success are reported for all 65 monkeys. Tests for normality were conducted using the Shapiro-Wilk test and normal Q–Q probability plots. We used the ‘lm’ function of the ‘stats’ package in R (R Core Team, 2016) to fit linear regression models using an information-theoretic approach on likelihood measures (AICc; Akaike, 1974) to identify the best predictors of training success (number of sessions) and number of trainers (1 or 2) required. The former was conducted for the females whose life history and behavioural data were available (n = 55). The predictor variables were age (continuous variable), number of adults in the group (continuous variable from 2 to 9), reproductive status (pregnant, dependent offspring, neither or both), dominance rank (high, medium, low), temperament (affiliative/cooperative, aggressive/uncooperative, predominantly neither), and early maternal separation (yes/no). We also included the null model in the analysis and used the ‘model.sel’ function in R to compare model fits. Given the limited window of time available for training (as few as 20 days for the more submissive females who were last to begin training), those monkeys who showed clear evidence of learning but failed to reach ‘criterion’ due to the shorter time available for them, were assigned a ceiling value of 50 sessions to retain them in the analysis (i.e. the maximum number of training days available to females within the training phase; for examples of use, see Ash and Buchanan-Smith, 2016, Held et al., 2006). We justify this on the basis that three monkeys who failed to reach criterion had performed well prior to the birth of their offspring midway through training; we have no reason to assume (based on the success rates of the cohort overall) that these monkeys would not have learnt the task otherwise.

## 3 Results

In total, 61 of the 65 monkeys who were approached for training, reached criterion for successful training to sit by a stationing tool (Table 3: 9/9 males; 52/56 females). Of the four females who did not train successfully, one we chose to discontinue training due to aggression towards her trainers and is therefore not included further in our analyses (henceforth n = 64). The other 3 females gave birth during training and failed to stay by their station for 30 s after 25 (6.25 h), 35 (8.75 h) and 40 (10 h) training sessions, respectively. Training was stopped for these animals, due to time constraints imposed by the start of the research program and they were assigned a session value of 50 for analysis. The successfully trained females reached criterion in an average of 7.4 training sessions (range 1–24). All nine males reached criterion for successful station training in two training sessions (and in addition they all learned to follow the command to “sit”).

**Table 3:** Group size, group composition (adults only) and training success.

Number of adults in group	Number of animals approached for training (M:F)	Number of animals successfully trained
1:8	1:8	1:7
1:8	1:8	1:7
1:8	1:6	1:6
1:9	1:6	1:5
1:5	1:5	1:5
1:5	1:5	1:5
1:7	1:4	1:4
2:3	2:3	2:3
0:6	0:5	0:5
0:9	0:4	0:3
0:2	0:2	0:2
**9:70**	**9:56**	**9:52**

Comparison of linear regression models (Table 4) revealed the only significant predictor of number of sessions required to train females was dominance rank (lm: F_(2.53)_ = 4.51, p = 0.038). High (n = 20) and mid (n = 22) ranking females reached criterion on average in 1 h 26 min (5.7 ± 1.06 sessions) and 1 h 52 min (7.45 ± 1.25 sessions), respectively, while low ranking females (n = 10) took on average 2 h 44 min ± 36 min (10.9 ± 2.39 sessions). All other factors (age, group size, reproductive status, temperament, and early maternal separation) failed to explain the data any better than the null model.

**Table 4:** Model comparison revealed dominance rank was the best predictor of the number of sessions to train.

Predictor variable	df	Log likelihood	AICc	delta	weight
Dominance rank	3	−208.98	424.4	0.00	0.52
Null model	2	−211.22	426.7	2.25	0.17

Forty of the female monkeys were successfully trained by a single trainer working alone. Model comparison showed that rank significantly explained the number of trainers required to successfully train a monkey (lm: F_(2.49)_ = 4.44, p = 0.01). A second trainer was useful in the training of lower ranked females, with 50% requiring 2 trainers present in order to reach success criteria; this was significantly different from high ranked females (t = 2.91, p = 0.005), who only needed a second trainer 5% of the time. Mid ranked females needed a second trainer in 27.27% of cases, which was not significantly different from high (t = 1.81, p = 0.08) or low (t = 1.49, p = 0.14) ranked females. All but one male were successfully trained with only one trainer present.

Four macaques, each from a separate group, would rattle their station tool so as to attract the trainer’s attention. We considered this to be an undesirable behaviour as it distracted the other monkeys which would be problematic during the planned research. We initially ignored the behaviour but it continued. When this behaviour occurred, we then ended the session for that animal and removed the station. In all cases, rattling decreased substantially to a point where it did not happen, or happened so infrequently that it was not deemed problematic, after two sessions.

## 4 Discussion

We present a PRT protocol and data for training rhesus macaques in breeding groups of up to nine adults (plus infants and juveniles) to approach and remain by individual stationing tools. We successfully trained 61 (out of an original 65 animals who were approached) during daily 15-min training sessions spread over a 12 week period. Following this protocol, training staff at similar facilities should expect to be able to train dominant male macaques within two daily training sessions; dominant and mid-ranking females within eight daily training sessions (2 h); and the lowest ranking females within 3 weeks or a month of daily training sessions (3–5 h). These results compare favourably with some previously published data. For example, Schapiro et al. (2003) successfully target trained 24/30 group housed adult rhesus macaques, reporting that the fastest animals trained within 55 min and the majority within 4 h. In that study, it was reported that dominant animals leaving their stations to take rewards from lower ranking individuals created the greatest time cost during training. In our study we tried to avoid this confound by targeting the dominant animals first. Fernstrom et al. (2009) successfully target trained 32/33 macaques, housed in groups of 2–3 individuals, in ∼15 × 30 min sessions. Our protocol for training macaques in social groups of up to 9 adults has a very comparable success rate to studies where trainers can focus on a couple of animals at a time.

When we initiated this study, we predicted that due to the strict hierarchy, generally aggressive temperament of rhesus macaques, and the presence of infants and juveniles, only the most dominant animals of each group would be successfully trained and therefore available for voluntary participation in the subsequent research program. However, we achieved a success rate far beyond our original expectations and 61 station-trained animals went on to take part in cognitive studies while freely moving in the social group. This demonstrates not only that the applied PRT methodology works, but also that it is possible for a single trainer to train multiple animals simultaneously and subsequently work with them during research procedures.

The only factor that predicted individual time to training success in our study was dominance rank. This is not surprising given the initial focus of the protocol on dominant animals, but is also in keeping with some previous studies which similarly found that lower ranked individuals take longer to train (e.g. Veeder et al., 2009; Wergård et al., 2016). This is most likely due to the fact that subordinate animals tend to be more prone to attack by dominants, and are typically more timid in approaching trainers or in remaining at their station, despite understanding the training contingencies. There was no effect of age, group size, reproductive status, temperament, or early maternal separation on time to train in our study. There were also no obvious predictors of failure to learn for the three females who did not reach criterion for successful training: one was high ranked and two were low ranked, and they were each housed in a different group (size range: 4–9 adults). The high ranked female (96 months, group size four adults) was particularly wary around people and showed little to no indication that the attempt at training (40 sessions) had made much impact although she would come forward if food was offered; the two low ranked females (131 months and 176 months, both in groups of 9 adults) were generally keen to train (35 and 40 sessions attempted, respectively) but were very wary of their group members and became more nervous after the birth of their infants. While they learned to approach their stations, they would not consistently remain at the station, holding or otherwise, for 30 s and therefore could not be considered ‘successfully trained’.

Thirty seconds was found to be a suitable time benchmark for training success, since macaques subsequently would remain at their station for longer periods during the later research phase. The cognitive testing (not presented here) often required the monkeys to be cooperative for periods of just over an hour, dependent on group size and willingness to work. Although the trained macaques did not sit at their station consistently for that whole period, we can report anecdotally that diversions from their stations were brief and animals could be encouraged quickly back to their stations if required. The training did ensure that the animals that did wander away rarely disturbed other macaques still at their station, which was our primary aim.

Throughout the sessions, the macaques were free to come and go as they chose; they were not constrained to the caged area. Indeed, it appeared that most stayed to watch the training of others. It is likely that this provided an opportunity for social learning (e.g. Perlman et al., 2010), and some monkeys appeared to show immediate understanding of the required behaviour at the start of their training. It was, therefore, essential to ensure that the first few monkeys in each group were properly trained and did not develop bad habits. We did find that a small number of macaques (n = 4), after most or all of their group had been trained, would rattle their station tool so as to attract the trainer’s attention. Ignoring this behaviour typically had no effect and it became necessary to retrain these animals to hold the stationing tool and wait for a reward. In some cases, it was necessary to end the session for that animal and remove its station – we found that the rattling behaviour would decrease substantially after two sessions in which this behaviour was ignored.

The biggest hindrance to training the females appeared to be the presence of newborn infants. Anecdotally, we observed some females became less willing to participate in the days after giving birth, but in some cases for up to several months afterwards. Mothers were often wary of the trainers if they came too close to their infants and could become mildly aggressive. This usually died down after a couple of sessions. We also had problems with older infants and juveniles snatching treats when we were offering them to adults, which could elicit aggression. We were not authorised to train the younger animals as some end users specify that they do not want previously trained animals. We hope that coordinated training protocols across facilities (and between breeders and end users) will eventually result, as the ease and benefits of working with animals in the social group are realised.

For some animals, it was helpful (although not essential) to have a second trainer present. This allowed one trainer to focus on a new trainee while the other trainer maintained the already trained animals. The methodology we have described here was suitable for one trainer to maintain, and we had success with it, with the majority of animals (n = 48) requiring only one trainer. Group size was not an explanatory variable, and we can report that, since this study, one trainer at the facility has single-handedly trained a group of 11 adults to station individually (Nightingale et al., 2015). However, for us to better access and attend to low-ranked females, a second trainer was useful, particularly during the early stages. By keeping the more dominant animals occupied, it was possible to focus one trainer’s attention on a low-ranked female, allowing the macaque to develop confidence in joining in without retribution from higher-ranked conspecifics. We recommend that a second trainer be used for this kind of training when one or more animals is particularly submissive to conspecifics.

The training presented here was successfully transferred to the subsequent cognitive testing phase of this study. Stationing was used to situate animals within each group in particular locations around the caging area, in order for one individual to be tested without other members of the group being able to view the visual stimuli directly. The training was used to primarily keep the macaque taking part in testing in the one location where we film performance, as well as keep other members of the group from interfering. An additional spin-off was care staff initiating their own training of the macaques. Stationing was an ideal starting point, given the small ratio of staff to macaques, and its usefulness for inspecting injuries and newborns. However, there were some difficulties to this transfer due to the monkeys’ prior relationship with care staff and restricted habituation opportunities. We encouraged further habituation sessions to help develop a more positive relationship and expanded these to visiting veterinary staff. Veterinarians commented that they noticed an attitude change from the macaques after two-three habituation sessions (Drs J. Willshire and J. Hemingway, personal communication). This reflects previous evidence that positive reinforcement can improve relationships between staff and animals (Bayne et al., 1993, Bloomsmith et al., 1997).

Throughout this paper, we use the term PRT to focus the reader’s attention on desired behaviours and their relationship to rewards. This is to avoid some of the misunderstandings that arise from common misuse of the learning theory terminology. For example, both positive reinforcement and negative punishment (see Table 1) were used in the protocol reported here. These terms relate to the appearance (‘positive’) or removal (‘negative’) of reward to increase the performance of desired behaviours (‘reinforcement’) or to decrease the performance of undesired behaviours (‘punishment’). It is important to note that the main focus of our training method was positive reinforcement but negative punishment was used in the case of the four females rattling their station tools (only 2 occurrences of this methodology were typically required to see a strong reduction in this behaviour). The important take-home message here is that we only manipulated the amount and frequency of *rewards* that animals received. Rewards activate dopamine systems in the primate brain and are linked to appetitive learning and seeking behaviour (Panksepp and Moskal, 2008); as we found the macaques to be highly food motivated, solving problems related to gaining access to food rewards should be, overall, an enriching experience. We avoided using negative reinforcement or positive punishment (Table 1), both of which use fear-eliciting stimuli to manipulate animals’ behaviour and are therefore likely to impact negatively on welfare (Laule and Whittaker, 2007, Prescott et al., 2005). Furthermore, our results here show that it is possible to train large numbers of group-housed macaques with minimal staff using only PRT.

Station training is generally considered to be the basic standard upon which other training protocols are built (Laule et al., 2003). Although it is not always possible to train every animal in a facility to cooperate in husbandry procedures, targeting a few key animals in each group should help to reduce stress and improve welfare. We hope that the protocol and data presented here will add to the existing literature and encourage others to take up PRT training of group-housed animals in facilities where this is not yet standard practice.

## 5 Conflict of interest

None.

## References

[bib0005] Akaike H. (1974). A new look at the statistical model identification. IEEE Trans. Autom. Control.

[bib0010] Ash H., Buchanan-Smith H.M. (2016). The long-term impact of infant rearing background on the affective state of adult common marmosets (*Callithrix jacchus*). Appl. Anim. Behav. Sci..

[bib0015] Bassett L., Buchanan-Smith H.M. (2007). Effects of predictability on the welfare of captive animals. Appl. Anim. Behav. Sci..

[bib0020] Bayne K., Dexter S., Strange D. (1993). The effects of food provisioning and human interaction on the behavioral well-being of rhesus monkeys (*Macaca mulatta*). Contemp. Top. (AALAS).

[bib0025] Bethell E.J., Semple S., Holmes A., MacLarnon A., Kemp C., Farningham D., Witham C., Wilding C., Hansen D., Arbuckle K. (2015). Attention bias a novel method to assess psychological wellbeing in group-housed macaques. Poster Presented at the NC3RS Primate Welfare Meeting.

[bib0030] Bloomsmith M.A., Laule G.E., Alford P.L., Thurston R.H. (1994). Using training to moderate chimpanzee aggression during feeding. Zoo Biol..

[bib0035] Bloomsmith M., Lambeth S., Stone A., Laule G. (1997). Comparing two types of human interaction as enrichment for chimpanzees. Am. J. Primatol..

[bib0040] Bloomsmith M.A., Stone A.M., Laule G.E. (1998). Positive reinforcement training to enhance the voluntary movement of group-housed chimpanzees. Zoo Biol..

[bib0045] Bloomsmith M.A., Marr M.J., Maple T.L. (2007). Addressing nonhuman primate behavioral problems through the application of operant conditioning: is the human treatment approach a useful model?. Appl. Anim. Behav. Sci..

[bib0050] Buchanan-Smith H.M., Badihi I. (2012). The psychology of control: effects of control over supplementary light on welfare of marmosets. Appl. Anim. Behav. Sci..

[bib0055] Capitanio J.P., Mendoza S.P., Lerche N.W., Mason W.A. (1998). Social stress results in altered glucocorticoid regulation and shorter survival in simian acquired immune deficiency syndrome. Proc. Natl. Acad. Sci. U. S. A..

[bib0060] Clay A.W., Bloomsmith M.A., Marr M.J., Maple T.L. (2009). Habituation and desensitization as methods for reducing fearful behavior in single housed rhesus macaques. Am. J. Primatol..

[bib0065] Coleman K.L., Pranger L., Maier A., Lambeth S.P., Perlman J.E., Thiele E., Schapiro S.J. (2008). Training rhesus macaques for venipuncture using positive reinforcement training techniques: a comparison with chimpanzees. J. Am. Assoc. Lab. Anim. Sci..

[bib0070] deWaal F.B.M., Luttrell L.M. (1985). The formal hierarchy of rhesus macaques: an investigation of the bared-teeth display. Ame. J. Primatol..

[bib0075] Fernstrom A.L., Fredlund H., Spangberg M., Westlund K. (2009). Positive reinforcement training in rhesus macaques – training progress as a result of training frequency. Am. J. Primatol..

[bib0080] Held S., Mason G., Mendl M. (2006). Maternal responsiveness of outdoor sows from first to fourth parties. Appl. Anim. Behav. Sci..

[bib0085] Honess P.E., Johnson P.J., Wolfensohn S.E. (2004). A study of behavioural responses of non-human primates to air transport and re-housing. Lab Anim..

[bib0090] Jackson W.M., Winnegrad R.L. (1988). Linearity in dominance hierarchies: a second look at the individual attributes model. Anim. Behav..

[bib0095] Jennings M., Prescott M.J., Buchanan-Smith H.M., Gamble M.R., Gore M., Hawkins P., Hubrecht R., Hudson S., Keeley J.R., Morris K., Morton D.B., Owen S., Pearce P.C., Robb D., Rumble R.J., Wolfensohn S., Buist D. (2009). Refinements in husbandry, care and common procedures for non-human primates. Ninth report of the BVAAWF/FRAME/RSPCA/UFAW joint working group on refinement. Lab. Anim..

[bib0100] LASA/MRC (2004). Key Considerations in the Breeding of Macaques and Marmosets for Scientific Purposes. https://www.mrc.ac.uk/research/policies-and-guidance-for-researchers/related-content/lasa-mrc-primate-breeding/.

[bib0105] Lambeth S.P., Hau J., Perlman J.E., Martino M., Schapiro S.J. (2006). Positive reinforcement training affects hematologic and serum chemistry values in captive chimpanzees (*Pan troglodytes*). Am. J. Primatol..

[bib0110] Laule G.E., Whittaker M.A., Brent L. (2001). The use of positive reinforcement techniques with chimpanzees for enhanced care and welfare. Care and Management of Captive Chimpanzees.

[bib0115] Laule G.E., Whittaker M.A. (2007). Enhancing nonhuman primate care and welfare through the use of positive reinforcement training. J. Appl. Anim. Welf. Sci..

[bib0120] Laule G.E., Thurston R.H., Alford P.L., Bloomsmith M.A. (1996). Training to reliably obtain blood and urine samples from a young diabetic chimpanzee (*Pan troglodytes*). Zoo Biol..

[bib0125] Laule G.E., Bloomsmith M.A., Schapiro S.J. (2003). The use of positive reinforcement training techniques to enhance the care, management, and welfare of primates in the laboratory. J. Appl. Anim. Welf. Sci..

[bib0130] NC3Rs (2006). NC3Rs Guidelines: Primate Accommodation, Care and Use. https://www.nc3rs.org.uk/non-human-primate-accommodation-care-and-use.

[bib0135] NC3Rs (2015). The Macaque Website. http://www.nc3rs.org.uk/macaques/.

[bib0140] Nightingale J., Merritt S., Kemp C. (2015). Station training of group housed rhesus macaques. Poster Presented at the NC3RS Primate Welfare Meeting.

[bib0145] Panksepp J., Moskal J., Elliot A.J. (2008). Dopamine and SEEKING: Subcortical reward systems and appetitive urges. Handbook of Approach and Avoidance Motivation.

[bib0150] Perlman J.E., Horner V., Bloosmith M.A., Lambeth S.P., Schapiro S.J., Lonsdorf E.V., Ross S.R., Matsuzawa T. (2010). Positive reinforcement training, social learning and chimpanzee welfare. The Mind of the Chimpanzee: Ecological and Experimental Perspectives.

[bib0155] Perlman J.E., Bloosmith M.A., Whittaker M.A., McMillan J.L., Minier D.E., McCowan B. (2012). Implementing positive reinforcement animal training programs at primate laboratories. Appl. Anim. Behav. Sci..

[bib0160] Prescott M.J. (2010). Ethics of primate use. Adv. Sci. Res..

[bib0165] Prescott M.J., Buchanan-Smith H.M. (2007). Training laboratory-housed non-human primates, part 1: A UK survey. Anim. Welf..

[bib0170] Prescott M.J., Bowell V.A., Buchanan-Smith H.M. (2005). Training laboratory-housed non-human primates, part 2: resources of developing and implementing training programmes. Anim. Technol. Welf..

[bib0175] Prescott M.J., Brown V.J., Flecknell P.A., Gaffan D., Garrod K., Lemon R.N., Watson J. (2010). Refinement of the use of food and fluid control as motivational tools for macaques used in behavioural neuroscience research: report of a Working Group of the NC3Rs. J. Neurosci. Methods.

[bib0180] Pryor K. (1999). Don’t Shoot the Dog: the New Art of Teaching and Training.

[bib0185] R Core Team (2016). R: A Language and Environment for Statistical Computing. https://www.R-project.org/.

[bib0190] Reinhardt V., Cowley D., Scheffler J., Verteinn R., Wegner F. (1990). Cortisol response of female rhesus monkeys to venipuncture in homecage versus venipuncture in restraint apparatus. J. Med. Primatol..

[bib0195] Reinhardt V. (1997). Training nonhuman primates to cooperate during handling procedures: a review. Anim. Technol..

[bib0200] Russell W.M.S., Burch R.L. (1959). The Principles of Humane Experimental Technique.

[bib0205] Schapiro S.J., Bloosmith M.A., Laule G.E. (2003). Positive reinforcement training as a technique to alter nonhuman primate behavior: quantitative assessments of effectiveness. J. Appl. Anim. Welf. Sci..

[bib0210] Schapiro S.J., Bloosmith M.A., Laule G.E. (2005). Training nonhuman primates to perform behaviors useful in biomedical research. Lab. Anim..

[bib0215] Shively C.A., Register T.C., Friedman D.P., Morgan T.M., Thompson J., Lanier T. (2005). Social stress-associated depression in adult female cynomolgus monkeys (*Macaca fascicularis*). Biol. Psychol..

[bib0220] Szott I. (2015). The Effect of Genotype on Attention Bias in Rhesus Macaques, *Macaca Mulatta*, as a Welfare Indicator.

[bib0225] Thatcher H. (2016). Testing the Emotional Value of Facial Stimuli Using Attention Bias in Rhesus Macaques, *Macaca Mulatta*.

[bib0230] Ward S.J., Melfi V. (2013). The implications of husbandry training on zoo animal response rates. Appl. Anim. Behav. Sci..

[bib0235] Wergård E.-M., Westlund K., Spångberg M., Fredlund H. (2016). Training success in group-housed long-tailed macaques (*Macaca fascicularis*) is better explained by personality than by social rank. Appl. Anim. Behav. Sci..

[bib0240] Westlund K. (2015). Training laboratory primates – benefits and techniques. Primate Biol..

[bib0245] Whittaker M. (2005). Applied problem solving to diminish abnormal behavior. Proceedings of the Seventh International Conference of Environmental Enrichment.

